# Hypoalbuminemia for the prediction of survival in patients with stage IVB cervical cancer

**DOI:** 10.1371/journal.pone.0273876

**Published:** 2022-09-02

**Authors:** Nobuhisa Yoshikawa, Masato Yoshihara, Satoshi Tamauchi, Yoshiki Ikeda, Akira Yokoi, Hiroaki Kajiyama

**Affiliations:** Department of Obstetrics and Gynecology, Nagoya University Graduate School of Medicine, Nagoya, Aichi, Japan; University Medical Center of Princeton at Plainsboro, UNITED STATES

## Abstract

We evaluated the prognostic significance of malnutrition in patients with metastatic cervical cancer. In this study, we retrospectively analyzed the cases of 43 patients with stage IVB (FIGO2018) cervical cancer treated at our institute from December 2004 to December 2017. We determined the correlation between clinicopathological characteristics and survival by performing univariate and multivariate analyses. The serum albumin value at diagnosis was used as an index of malnutrition. The median follow-up period was 16.4 months (range, 0.9–91.4 months). On Kaplan-Meier analysis, the 1- and 2-year overall survival (OS) rates for all patients were 61.6% and 48.6%, respectively. The optimal serum albumin for predicting 1-year survival was 3.3 g/dL, as determined by the receiver operating characteristic curve to maximize the area under the curve. The OS of the patients with albumin >3.3 g/dL (n = 28) was significantly better than that of the patients with albumin ≤3.3 g/dL (n = 15) (p = 0.004). The univariate and multivariate analyses revealed that pretreatment serum albumin and mode of primary treatment were significantly associated with survival in patients with stage IVB cervical cancer. Hypoalbuminemia was an unfavorable prognostic factor for patients with metastatic cervical cancer.

## Introduction

There were approx. 570,000 new cases and over 310,000 deaths of cervical cancer worldwide in 2018 [1]. Despite the introduction of the human papillomavirus (HPV) vaccine into national immunization programs and a high cover rate of Pap screening for the early detection of cervical cancer, this cancer remains a fatal disease for women [2]. Stage IVB (metastatic cervical cancer) with distant organ metastasis accounts for 5.5%–8.4% of all cervical cancer stages, and the prognosis is remarkably severe [3–6]. Systemic chemotherapy, palliative radiotherapy, and the best supportive care are the main treatments for metastatic cervical cancer, except for the minority of patients who respond to local treatment [3]. Because metastatic cervical cancer is relatively rare, studies are often designed to include not only metastatic cervical cancer but also persistent or recurrent cervical cancer after primary treatment [7]. However, metastatic cervical cancer is entirely different from persistent/recurrent cervical cancer after primary treatment in terms of sensitivity to treatment based on prior treatment. These two forms of cervical cancer should therefore be evaluated separately.

The FIGO staging system was revised in 2018 and now includes not only clinical findings; the system also incorporates surgical pathological findings and advanced imaging [8]. The frequency of stage IVB patients thus increased with the update of the staging system from version 2009 to version 2018 [9]. This has resulted in the inclusion of a broader group of patients at stage IVB, and treatment should be individualized based on the patient’s general condition and metastatic status. However, there is no precise indicator that can point to the best treatment option.

Compared to early-stage cancer, advanced cancers’ current treatments present increased risks of side effects and treatment-related deaths. The identification of prognostic markers based on the individual patient’s general condition and nutritional status is thus essential for selecting the optimal treatment for patients with advanced cancer, to maximize the possibility of a cure and minimizing side effects. It is especially desirable to be able to predict patients’ survival and serious complications based on existing blood samples and imaging findings, which are valuable because of their versatility and immediate clinical applicability. For example, a patient’s performance status (PS) is frequently used as a representative index reflecting the patient’s general condition [10,11]. Malnutrition and sarcopenia are often observed in patients with advanced cancer, and the severity of these two factors has been reported to correlate strongly with subsequent life expectancy. For example, the prognostic nutritional index (PNI), which is calculated using serum albumin levels and lymphocyte counts, was reported to correlate with long-term prognosis and perioperative complications in patients with various carcinomas [12–15].

There have been several studies of cervical cancer that used the PNI, but there are relatively few reports on prognostic factors for metastatic cervical cancer. We recently demonstrated that patients with metastatic cervical cancer who are sarcopenic at diagnosis have a poor prognosis [16]. To the best of our knowledge, no index can predict the prognosis of patients with metastatic cervical cancer in advance and thus contribute to the appropriate treatment options. We conducted the present study to determine the prognostic impact of the albumin value in patients with metastatic cervical cancer.

## Methods

### Study population

We retrospectively analyzed the cases (medical records) of patients with cervical cancer who were diagnosed at our hospital during the period December 2004 to December 2017. The study was conducted according to the guidelines of the Declaration of Helsinki, and approved by the Institutional Review Board of Nagoya University Hospital (2013–0078). The institutional review board issued a waiver for patients’ written informed consent because the study design was retrospective. Of the total of 838 patients with cervical cancer diagnosed during the study period, 45 (5.4%) were diagnosed with primary cervical cancer with organ metastasis by physical examination and/or imaging. Two cases with insufficient data were excluded; a final total of 43 patients’ cases were included. Patients with only para-aortic metastasis were excluded from this study.

### Data extraction

We extracted the data from medical records regarding factors that may affect survival outcomes: age at diagnosis, body mass index (BMI) before treatment, type of histology, clinical TNM classification, mode of primary treatment (concurrent chemoradiotherapy [CCRT] or others), and the pre-treatment laboratory values including hemoglobin (Hb), total white blood cell count (TWBC), platelet count (Plt), albumin, and squamous cell carcinoma (SCC) antigen. In all cases, MRI and CT were performed for the evaluation of local progression and systemic metastases.

### Treatment

The treatment strategy for each patient was determined by a conference consisting of several gynecologic oncologists and radiologists. CCRT was principally selected as the primary treatment to control locoregional tumors, even for patients with stage IVB cervical cancer. Another option such as radiation, systemic chemotherapy, or best supportive care was chosen as the initial treatment only when the patient desired a treatment other than CCRT, or when a treatment option other than CCRT was desired due to the patient’s severe renal dysfunction or poor general condition based on PS. The CCRT consisted of external beam radiotherapy (EBRT) and intracavitary brachytherapy (ICBT). The standard EBRT dose was up to 50.4 Gy and was given as 1.8 Gy per dose 5 days/week. At our hospital, the effectiveness of EBRT was evaluated by MRI at the time of irradiation of approx. 36–39.6 Gy and ICBT was performed using a remote after-loading system with a Co60 source for patients whose cancer was considered to be locally controllable. Concurrent chemotherapy was administered the same day as the beginning of the EBRT and included 3–5 cycles of 5-fluorouracil (700 mg/m^2^ for 4 consecutive days)/cisplatin (70 mg/m^2^ on the first day) every 3 weeks. Other treatment options were RT alone, a hysterectomy followed by adjuvant CCRT or systemic chemotherapy, primary systemic chemotherapy, and palliative support.

### Follow-up and definition of survival

A post-treatment follow-up was performed every 1–2 months during the first year, and less frequently after the second year if there were no signs of recurrence. The routine follow-up included a physical examination, transvaginal ultrasonography, and blood tests that included tumor markers. The follow-up period was defined as the date of treatment initiation to the date of last confirmed survival or death. CT was performed at least every 3–6 months as a rule. The diagnosis of recurrence was based on CT images. Overall survival (OS) was defined as the time from the date of the patient’s first treatment to the date of death for any reason or the patient’s last visit to our hospital. Progression-free survival (PFS) was defined as the time from the date of the patient’s first treatment to disease progression or death from any cause.

### Statistical analyses

The statistical analyses were performed with JMP 14.0 software (SAS, Cary, NC). Based on a receiver operating characteristic (ROC) curve analysis, the cutoff value was determined to be the value that could detect survival at 1 year after the start of treatment with the highest sensitivity and specificity. This value was determined to maximize the area under the ROC curve. Patient characteristics were compared between groups using the qualitative chi-square test and the quantitative T-test or Mann-Whitney U-test. The Kaplan-Meier method was used for the survival analysis. The p-value for comparison of survival rates between groups was calculated by log-rank test. The univariate analysis of survival was performed using the univariate Cox regression analysis. Multivariate analysis was performed using only the variables that were significantly related to survival in the univariate analysis by the Cox proportional hazard model to minimize the effect of any factors confounding bias on survival. A p-value <0.05 was considered significant.

## Results

**Fig 1:**
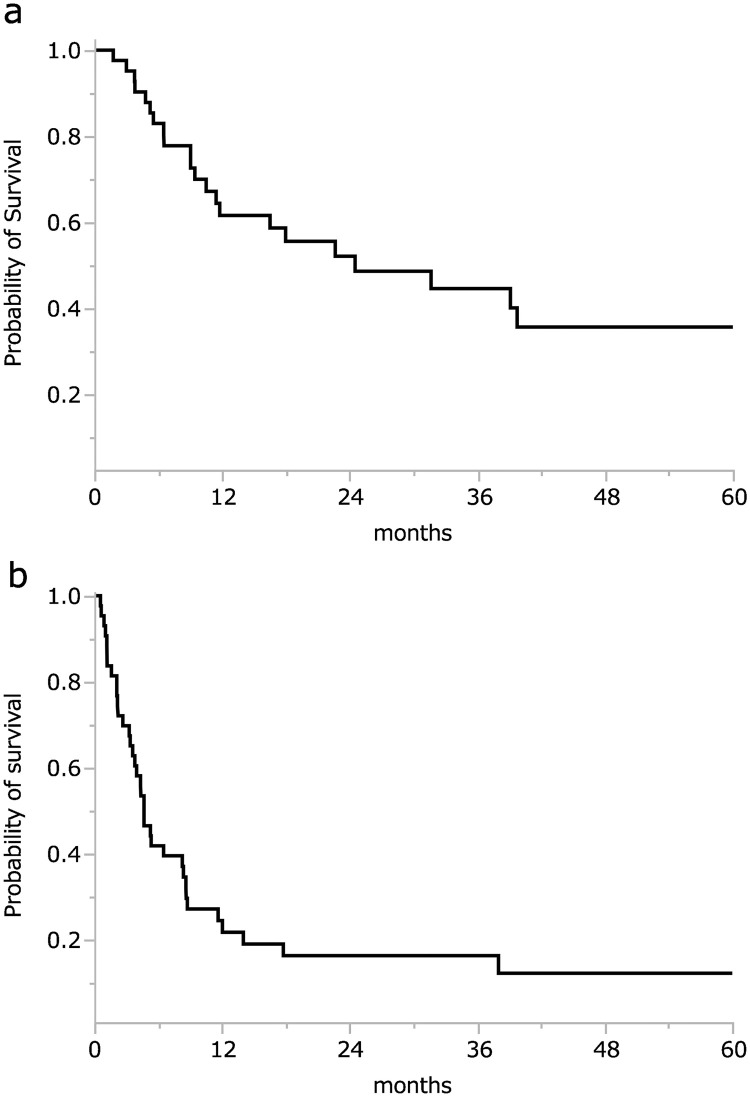
Kaplan-Meier curves show overall survival (OS) and progression-free survival (PFS) in all patients. The 1- and 2-year OS rates for all patients were 61.6% and 48.6%, respectively **(a)**. The 1- and 2-year PFS rates were 25.0% and 16.7%, respectively **(b)**.

A total of 43 patients were enrolled. The median follow-up period was 16.4 months (range, 0.9–91.4 months). The Kaplan-Meier analysis results revealed that the 1- and 2-year OS rates for all patients were 61.6% and 48.6%, respectively (Fig 1a). The 1- and 2-year PFS rates were 25.0% and 16.7% (Fig 1b).

**Fig 2:**
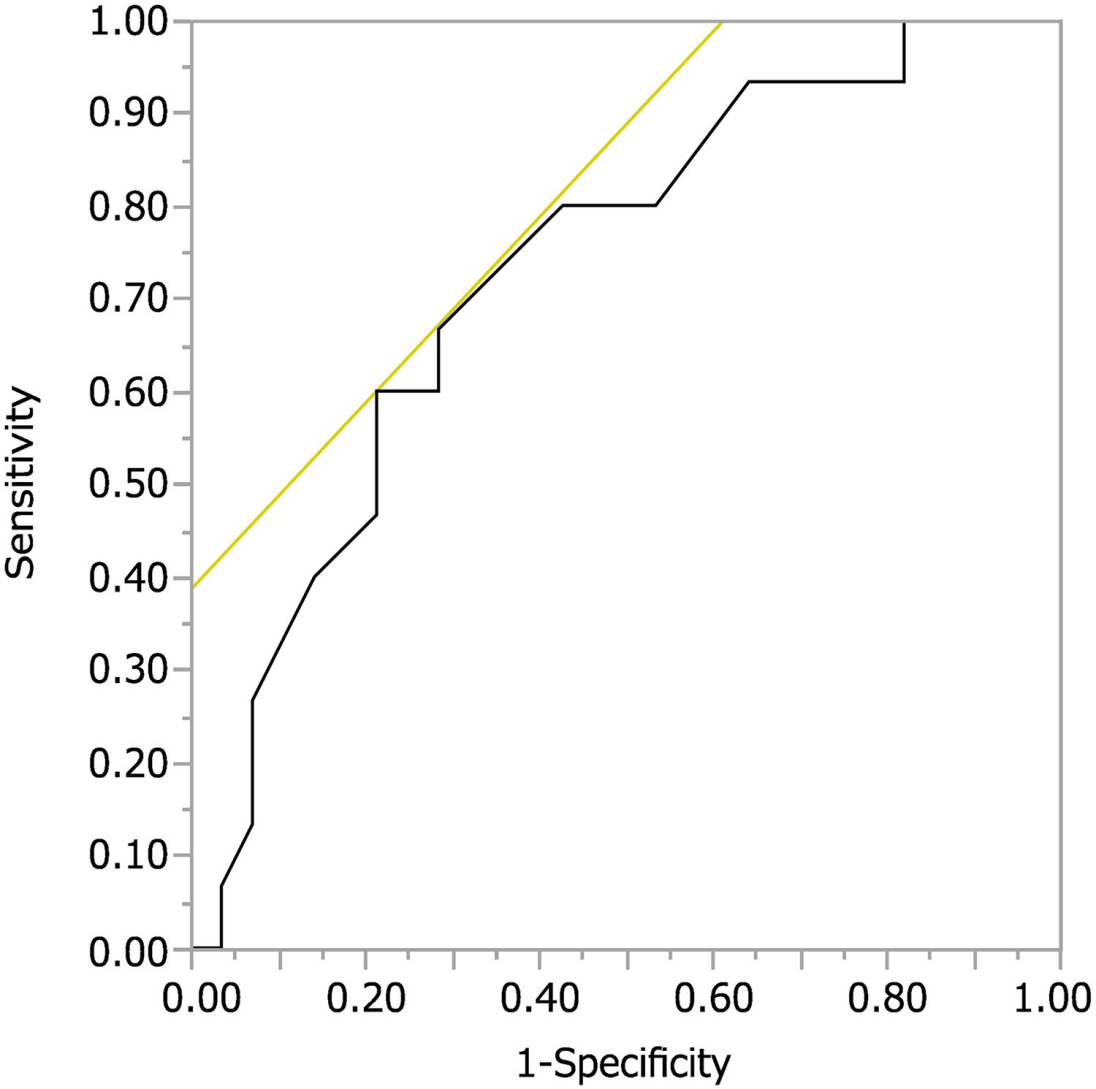
Receiver operating characteristic curve analysis of pretreatment serum albumin. The maximum area under the curve (AUC) was 0.729 for pretreatment serum albumin.

First, in our effort to identify one or more novel biomarkers related to long-term survival in this patient population, we performed a ROC analysis to compare four pre-treatment laboratory parameters: Hb, TWBC count, Plt, and albumin. The C-reactive protein (CRP) value was excluded from the analysis due to many missing data. We obtained the ROC curves to identify the optimal value of each valuable for predicting survival and an ROC curve for 1-year survival. The maximum area under the curve (AUC) was 0.729 for albumin, and the optimal cutoff value for predicting 1-year survival was 3.3 g/dL (Fig 2). The determined optimal cutoff value for albumin had 60.0% sensitivity and 78.6% specificity.

We next divided the patients into two groups by using the albumin cutoff value of 3.3 g/dL: the albumin-low group (albumin ≤3.3 g/dL, n = 15) and the albumin-high group (albumin >3.3, n = 28). The clinicopathologic characteristics of all patients stratified into the two groups are shown in Table 1. Pre-treatment characteristics were similar between the two groups with the exception of the rate of locoregional extension of the primary tumor (cT) and the primary treatment. Pre-treatment hypoalbuminemia was significantly associated with severe local progression of the primary tumor.

**Table 1:** Patients’ clinical characteristics are stratified by albumin-low and albumin-high groups.

	All case		Alb-low		Alb-high		P-value
	n	%	n	%	n	%	
Total patients	43	100	15	34.9	28	65.1	
**Follow-up time, months (median, range)**	16.4 (0.9–91.4)		6.5 (0.9–49.4)		16.4 (0.9–91.4)		
**Age, years (median, range)**	58.9 (28.2–84.0)						
≤ 60	23	53.5	7	46.7	16	57.1	0.511
> 60	20	46.5	8	53.3	12	42.9	
**BMI (mean ± SD)**	20.3 ± 3.3						
≤ 22	30	69.8	13	86.7	17	60.7	0.065
> 22	13	30.2	2	13.3	11	39.3	
**Histology**							
Squamous cell carcinoma	28	65.1	10	66.7	18	64.3	0.875
Adenocarcinoma or others	15	34.9	5	33.3	10	35.7	
**cT**							
1–2	17	39.4	0	0	17	60.7	<0.001
3–4	26	60.5	15	100	11	39.3	
**N**							
0	9	20.9	1	6.7	8	28.6	0.070
1	34	79.1	14	93.3	20	71.4	
**M**							
lung only	16	37.2	4	26.7	12	42.9	0.288
others	27	62.8	11	73.3	16	57.1	
**Primary Treatment**							
CCRT	32	74.4	8	53.3	23	82.1	0.047
Others	11	25.6	7	46.7	5	17.9	
**SCC antigen, ng/mL** **(median, range)**	7.8, 0.6 − 465.6		13.3, 1 − 465.6		6.75, 0.6 − 130		0.004
**TWBC, 10** ^**3**^ **/μL (mean ± SD)**	9.50 ± 3.95		11.58 ± 5.23		8.39 ± 2.54		0.010
**Hemoglobin, g/dL (mean ± SD)**	10.62 ± 2.15		9.16 ± 1.59		11.40 ± 2.02		<0.001
**Platelet, 10** ^**3**^ **/μL (mean ± SD)**	340.90 ± 117.46		394.33 ± 102.76		312.28 ± 116.41		0.027
**Albumin, g/dL (mean ± SD)**	3.51 ± 0.81						


BMI: Body mass index, CCRT: Concurrent chemoradiotherapy, TWBC: Total white blood cell, SD: Standard deviation.

Table 2 summarizes the results of the univariate and multivariate analyses of clinicopathologic variables that were potentially related to the patients’ OS and PFS. Univariate analysis identified pretreatment albumin levels and primary treatment as variables involved in OS. For all 43 patients, albumin ≤3.3 was significantly associated with shorter OS (hazard ratio [HR] 3.26, 95% confidence interval [CI]: 1.35–7.67; p = 0.009). Patients treated with CCRT had significantly longer survival compared to the patients who received other treatments (HR 3.26, 95%CI: 1.22–7.96; p = 0.020). For PFS, none of the variables showed a significant correlation with survival. The multivariate analysis also revealed that the following two factors were significantly associated with a favorable prognosis in OS: albumin >3.3 (HR 3.40, 95%CI: 1.38–8.21; p = 0.008) and CCRT as the primary treatment (HR 3.46, 95%CI: 1.24–8.87; p = 0.020).

**Table 2:** Results of the Cox regression models for overall survival and progression-free survival.

Variable	OS				PFS			
	Univariate		Multivariate		Univariate		Multivariate	
	HR (95% CI)	P-value	HR (95% CI)	P-value	HR (95% CI)	P-value	HR (95% CI)	P-value
Age at diagnosis (>60 vs. ≤60)	1.07 (0.45– 2.50)	0.859			0.94 (0.48– 1.83)	0.870		
Preoperative BMI (>22 vs. ≤22)	0.88 (0.31– 2.14)	0.799			1.42 (0.68– 2.81)	0.334		
Histology (Adenocarcinoma or others vs. Squamous cell carcinoma)	0.94 (0.36– 2.22)	0.904			1.44 (0.71– 2.82)	0.292		
cT (3–4 vs. 1–2)	1.69 (0.72– 4.23)	0.225			1.36 (0.70– 2.73)	0.357		
N (1 vs. 0)	1.10 (0.45– 3.08)	0.826			0.81 (0.38– 1.91)	0.614		
M (lung metastasis only vs. others)	1.39 (0.57– 3.20)	0.448			1.79 (0.83– 3.75)	0.131		
Primary Treatment (others vs. CCRT)	3.26 (1.22– 7.96)	0.020	3.46 (1.24– 8.87)	0.020	1.55 (0.72– 3.09)	0.244		
SCC antigen (>10 vs. ≤10 or NA)	1.59 (0.68– 3.77)	0.278			1.07 (0.54– 2.08)	0.827		
TWBC	0.98 (0.82– 1.15)	0.881			1.09 (0.97– 1.21)	0.120		
Hemoglobin	0.91 (0.77– 1.08)	0.314			0.97 (0.84– 1.12)	0.734		
Platelet	0.99 (0.99– 1.00)	0.840			0.99 (0.99– 1.00)	0.913		
Albumin (≤3.3 vs. >3.3)	3.26 (1.35– 7.67)	0.009	3.40 (1.38– 8.21)	0.008	0.79 (0.40– 1.61)	0.515		

BMI: Body mass index, CCRT: Concurrent chemoradiotherapy, TWBC: Total white blood cell, SD: Standard deviation.

**Fig 3:**
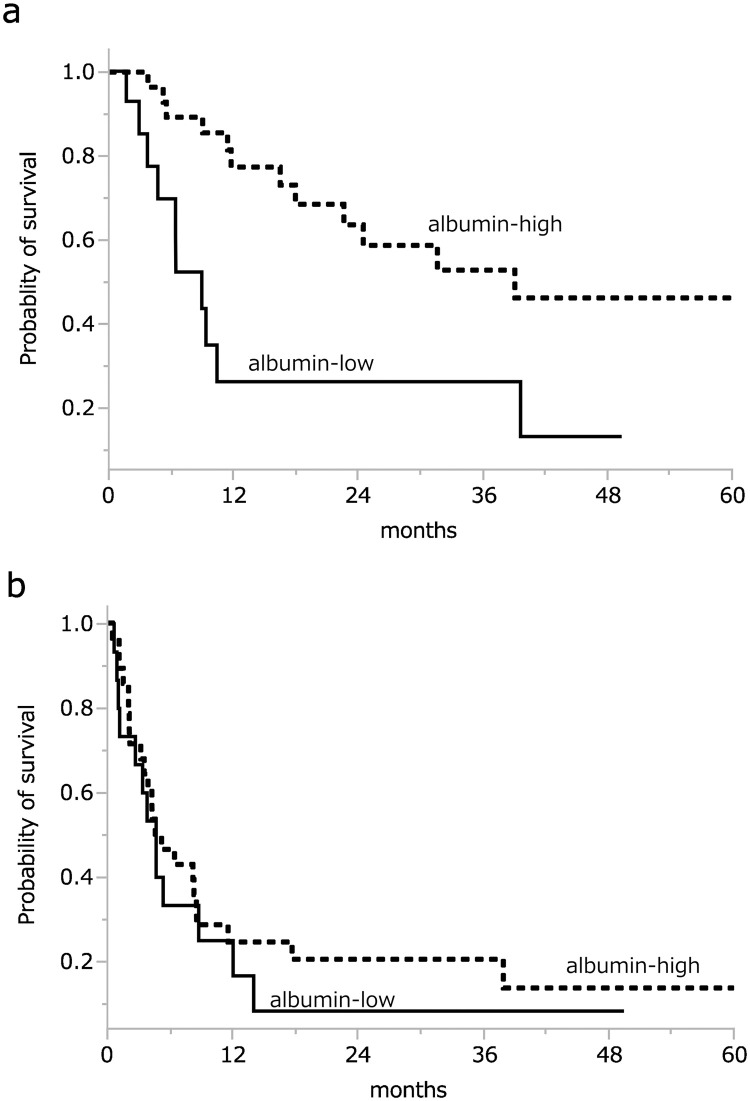
Kaplan-Meier curves show overall survival (OS) and progression-free survival (PFS) stratified by pretreatment serum albumin. The 1-year OS rate in the albumin-low group (―) was 26.1%, and that in the albumin-high group (----) was 77.4% (p<0.05). The 1-year PFS rate in the albumin-low group (―) was 25.0%, and that in the albumin-high group (----) was 24.4% (p = 0.507).

To clarify the prognostic impact of pre-treatment hypoalbuminemia, we performed a Kaplan-Meier survival analysis and log-rank test: the 1-year OS rate in the albumin-low group was 26.1%, and that in the albumin-high group was 77.4% (p = 0.004) (Fig 3a). In contrast, there was no significant difference in PFS between the two groups: the 1-year PFS rate in the albumin-low group was 25.0%, and that in the albumin-high group was 24.4% (p = 0.507) (Fig 3b). To examine whether the pre-treatment albumin value >3.3 is useful for selecting the patient’s primary treatment, we conducted a survival analysis between patients who underwent CCRT and those treated with another therapy in the albumin-high group. In the albumin-high group, the patients treated with CCRT had longer survival than those who received another treatment, but the difference was not significant (p = 0.129).

## Discussion

We retrospectively analyzed the cases of patients with metastatic cervical cancer to investigate the significance of pre-treatment albumin for the prediction of overall survival. The results of a multivariate analysis demonstrated that the presence of hypoalbuminemia predicted a short survival time in this patient cohort. Our analyses also revealed that the patients with albumin >3.3 g/dL who underwent CCRT were likely to survive longer than the patients who underwent other treatments. Pre-treatment albumin is a useful biomarker to identify patients for whom CCRT can be expected to prolong survival.

Similar to the reports on other malignancies, the present results suggest that there is a strong association between pretreatment serum albumin and the prognosis of cervical cancer patients [12,17]. Although the significance of hypoalbuminemia in cervical cancer has not been clear, we found a correlation when limited to metastatic cervical cancer. Since stage IVB accounts for less than 10% of cervical cancer, it was inferred that serum albumin is unlikely to be revealed its function as a prognostic factor. Hypoalbuminemia reflects a state of increased catabolism due to tumor-derived cytokines and tumor progression itself. In patients with malignant tumors, the serum albumin level tends to decrease because of malnutrition and systemic inflammatory responses. The present analysis demonstrated a significant negative correlation between albumin and CRP, which reflects systemic inflammation. Cachexia accompanied by hypoalbuminemia is a complex condition involving the tumor, the host response to the tumor, and anticancer therapy. Hypoalbuminemia has been reported to be associated with low quality of life (QOL) and an increased risk of adverse effects induced by chemotherapy during the treatment for malignancies [18,19]. A patient’s pre-treatment serum albumin level may thus be a useful tool for estimating the patient’s tolerance to treatment intensity and survival prognosis. It may also be useful to consider this value about nutritional interventions to elevate a patient’s albumin.

We recently reported that sarcopenia at diagnosis is a prognostic factor in patients with metastatic cervical cancer [16]. We also reported the prognostic value of the neutrophil-to-lymphocyte ratio in early-stage ovarian clear-cell carcinoma [20]. There have also been reports that sarcopenia, cachexia, and malnutrition in gynecological malignancies are useful in predicting prognosis [16,21,22]. In the case of metastatic cervical cancer, it is often difficult to decide whether to choose radiotherapy, chemotherapy, chemoradiotherapy or the best supportive care as the primary treatment. The performance status according to the Eastern Cooperative Oncology Group is often taken into consideration, but this PS is sometimes felt to be less objective. The preoperative serum albumin is a more objective biomarker that adequately reflects cancer cachexia.

We also observed that CCRT as the primary treatment was efficient for improving the OS of the patients with distant metastases (HR 3.40, 95%CI: 1.38–8.21). Our hospital’s policy was to choose CCRT as a general rule when the patient’s symptoms due to physical weakness or distant metastasis were not severe. Our finding that CCRT was a favorable prognostic factor in this study may thus have been affected by selection bias. Past studies of the 5-year survival rate of stage IVB cervical cancer have reported rates of 5.5%–8.4% [3–6]. Although it is difficult to make a simple comparison, the 5-year survival rate in this study was 35.7%, which is better than previously reported. This is due to the high local control rate that 12 out of 32 patients treated with CCRT (37.5%) achieved a complete response in the locoregional tumor. This suggests the potential of CCRT for stage IVB cervical cancer with distant metastasis. Furthermore, our results showed that two variables including primary therapy and pretreatment albumin are significantly related to OS, but not PFS. This may be due to the fact that the very wide range of general conditions and cancer aggressiveness in individual cases can lead to discrepancies between PFS, which better reflects treatment response, and OS, which better reflects overall general condition.

There are some study limitations to consider. It was a retrospective analysis, and bias and confounding factors were thus present. The small sample size (n = 43) could have resulted in a type-II error. For example, known poor prognostic factors for cervical cancer, such as older age and locoregional progression (cT), were not significantly correlated with prognosis in the current study [23]. This may be due to the the small sample size of this study as well as the possibility that the prognostic significance of age and locoregional progression in stage IVB is unclear. In clinical practice, patients with organ metastatic cervical cancer are very diverse, and it is necessary to take into account various parameters such as the mode of metastasis and the patient’s general condition to determine the optimal treatment. Our findings are therefore merely hypothesis-generating, but our observation that albumin not only predicts the prognosis of patients with organ-metastatic cervical cancer but also indicates the possibility of more effective treatment for some patients with a promising long-term prognosis is of value. In other words, even in patients with organ-metastatic cervical cancer, if the albumin level is >3.3, CCRT may be more effective in prolonging life as the initial treatment. Prospective clinical trials in patients with organ metastatic cervical cancer using an albumin-based risk assessment are expected in the future.

In conclusion, in the present patient series, the malnourished patients (albumin ≤3.3 g/dL) with organ metastatic cervical cancer had shorter survival than those with albumin >3.3 g/dL.

### Institutional review board statement

The study was conducted according to the guidelines of the Declaration of Helsinki and approved by the Institutional Review Board of Nagoya University Hospital (2013–0078). Patient consent was waived because data collection was retrospective.
